# The Influence of Aging on Outcomes in Patients Undergoing Percutaneous Nephrolithotomy for Complete Staghorn Stones: A Retrospective Comparative Study

**DOI:** 10.7759/cureus.20001

**Published:** 2021-11-29

**Authors:** Murat Sahan, Serkan Yarımoğlu, Metin Savun, Onur Erdemoglu, Tansu Degirmenci

**Affiliations:** 1 Urology, University of Health Sciences İzmir Bozyaka Education and Research Hospital, Izmir, TUR; 2 Urology, Basaksehir Cam and Sakura City Hospital, Istanbul, TUR

**Keywords:** urolithiasis, age, clavien scoring system, staghorn renal stone, percutaneous nephrolithotomy

## Abstract

Objective

In this study, we aimed to evaluate the effect of age on the success and complications of percutaneous nephrolithotomy (PCNL) for complete staghorn renal stones.

Materials and methods

The files of 182 patients who underwent single-access PCNL for complete staghorn renal stones between 2012 and 2017 were retrospectively analyzed. The patients were divided into two groups according to their age: those aged <65 years were defined as Group-1 and those aged ≥65 years as Group-2. The demographic characteristics and perioperative and postoperative results were compared between the two groups.

Results

Among the patients with complete staghorn renal stones, 139 were in Group-1 and 43 were in Group-2. The mean age of the patients was 43.9 ±10.6 years in Group-1 and 67.8 ±2.1 years in Group-2 (p=0.001). The rate of hemoglobin drop was significantly higher in Group-1 (p=0.001). However, the blood transfusion rate was higher in Group-2 than in Group-1 (18.6% vs. 7.2%, respectively). The stone-free rate was 54.7% in Group-1 and 67.4% in Group-2 (p=0.139). As for the evaluation of the overall complication rates, 34.5% of the patients in Group-1 and 46.5% of those in Group-2 developed complications (p=0.206).

According to the Clavien scoring system, the rate of minor complications was found to be 22.3% in Group-1 and 41.9% in Group-2, and the difference was statistically significant (p=0.012). The major complication rates were determined as 4.7% and 12.2% for Group-1 and Group-2, respectively (p=0.155). The number of patients with Clavien grade-2 complications was significantly higher in the elderly patient group (p=0.019).

Conclusions

Based on our findings, PCNL is an effective and safe method in the treatment of complete staghorn stones in elderly patients.

## Introduction

Individuals aged 65 years and over are generally defined as the "elderly population" [1]. The decrease in birth rates, as well as the prolongation of life expectancy at birth and advanced age, have led to a relative increase in the elderly population and its share in the total population, especially in developed and developing countries. As a result of this increase, the number of elderly patients seeking treatment due to urinary system stones is on the rise. Staghorn stones are of particular concern in this age group because the management of such stones may require lengthy and multiple procedures and has a high complication rate.

Staghorn stones are branching and usually infected stones that cover a large part of the collecting system [2]. Failure to achieve stone-free status may lead to the complete loss of function and sepsis in the kidney due to the destruction of the renal parenchyma. Guidelines recommend percutaneous nephrolithotomy (PCNL) as the standard treatment modality for renal stones >2 cm [3]. The overall success of PCNL is up to 96.1% [4]. However, since multiple percutaneous accesses may be required to remove all stone branches in staghorn stones, it is very difficult to achieve stone-free status in these patients [5]. Therefore, as reported in previous studies, stone-free rates in staghorn stones can be as low as 56.9% [6]. In addition to these low stone-free rates after PCNL, staghorn stones also have high complication rates. In a prospective randomized study, the intraoperative complication rate of PCNL in the treatment of staghorn stones was found to be 16.3%, and the postoperative major complication rate was determined to be 18.6% [7].

There are concerns related to the safe applicability of PCNL in geriatric patients with bleeding tendencies and low cardiopulmonary performance. In light of this, we aimed to compare the results between elderly and younger patients who underwent PCNL for complete staghorn stones.

A preliminary version of this manuscript was made available on Authorea as a preprint on June 21, 2021, and assigned a DOI (10.22541/au.162430432.25582170/v1). However, the manuscript or its contents have not been previously published.

## Materials and methods

This retrospective study was conducted among patients who had undergone PCNL for complete staghorn stones between April 2012 and January 2017. Patients with a skeletal deformity, congenital kidney anomalies, coagulopathy (chronic diseases like liver disease, vitamin K deficiency, disseminated intravascular coagulation, factor deficiency, thrombocytopenia, von Willebrand disease, hemophilia, etc.), and solitary kidneys, cases requiring multiple accesses, and patients without complete staghorn stones were excluded from the study. A total of 182 consecutive patients with complete staghorn stones were selected and divided into two groups according to patient age: those aged <65 years were defined as Group-1 and those aged ≥65 years as Group-2. The demographic, perioperative, and postoperative data were compared between these two groups.

All the patients were evaluated preoperatively using standard non-contrast abdominal CT. The patients’ demographic and preoperative characteristics, including operation side and history, stone burden, gender, metabolic syndrome [there were at least two illnesses (diabetes mellitus, hypertension, hyperlipidemia, and obesity)], and stone density were recorded. In addition, intraoperative and postoperative results (operation and fluoroscopy time, nephroscopy time, calyx accessed, complications, and stone-free status) were examined. Postoperative complications were evaluated according to the Clavien scoring system [8].

After the patients returned a negative urine culture, and their laboratory tests like complete blood count (CBC), activated partial thromboplastin time (aPTT), and prothrombin time (PT) tests were within the normal reference range, they were taken for the operation. Antiplatelet or anticoagulant medication was stopped before the procedure according to clinicians’ suggestion.

The stone burden was calculated in square millimeters in all patients (length x width x π x 0.25, where 3.14 was taken as the mathematical constant) [9]. For staghorn stones, this calculation was performed separately for each calyceal stone and the sum of all values was accepted as the result. All PCNL operations were performed by experienced urologists. Success was defined as a complete stoneless state or the detection of <4-mm stones on control CT performed at the first postoperative monthly follow-up.

Operation technique

After placing five or six F ureter catheters under general anesthesia, subcostal or intercostal access was achieved in all patients with an 18-gauge needle with fluoroscopic guidance in the prone position depending on the location of the stone and the anatomy of the kidney. The entry site was dilated up to 30 Fr using Amplatz dilators, and the collecting system was entered with a nephroscope. Lithotripsy was performed with a pneumatic lithotriptor (Vibrolith; Elmed, Ankara, Turkey). A 14-F nephrostomy tube was inserted postoperatively in all patients.

Statistical analysis

The SPSS Statistics version 22 (IBM, Armonk, NY) software package was used to analyze the data. The independent-samples t-test, chi-square test, and Fisher’s exact test were used to compare the two groups. Quantitative data were expressed as mean ±standard deviation in tables. Categorical data were presented as numbers (frequency) and percentages (%). Data were analyzed at a 95% confidence level, and a p-value of less than 0.05 was considered statistically significant.

The study was approved by the Institutional Ethical Board of the Health Sciences University, Bozyaka Education and Research Hospital, Izmir, Turkey (meeting/decision no. 2021-165 dated September 29, 2021). All procedures adhered to the ethical guidelines of the Declaration of Helsinki and its amendments. Written permission was obtained before the procedure and for the use of clinical details (without disclosing patients' identities) for academic purposes. All original data reported in this study can be made available on request.

## Results

There were 139 patients in Group-1 and 43 patients in Group-2. The mean ages of the patients were 43.9 ±10.6 and 67.8 ± 2.1 years in Group-1 and Group-2, respectively (p=0.001). The mean stone size was 899 ±292 mm^2^ in Group-1 and 1,008 ±208 mm^2^ in Group-2 (p=0.736). Also, the operation side, metabolic syndrome, the mean body mass index, and the Hounsfield units were comparable between the two groups (p=0.386, p=0.443, p=0.692, and p=0.084, respectively). Gender distribution and history of previous renal stone surgeries were similar in both groups (p=0.577 and p=0.966, respectively). The similarity of demographic data and stone characteristics in the two groups emphasized the effect of age. As a result, the effect of age on stone-free status and complications in the two groups was deemed comparable (Table 1).

**Table 1:** Comparison of demographic data and stone characteristics between the study groups *Mean ±standard deviation

Variables	Group-1 (<65 years)	Group-2 (≥65 years)	P-value
Number of patients	139	43	
Gender (female/male)	42/97	15/28	0.577
Age (years)*	43.9 ±10.6	67.8 ±2.1	0.001
Body mass index (kg/m^2^)*	26.7 ±4.3	27.5 ±5.7	0.692
Metabolic syndrome	6 (4.3%)	3 (7.0%)	0.443
History of operation	48 (34.5%)	15 (34.9%)	0.966
Stone size (mm^2^)*	899 ±292	1,008 ±208	0.736
Hounsfield unit (HU)*	1,097 ±345	983 ±304	0.084
Operation side (right/left)	66/73	17/26	0.386

When we examined the operative data, we determined that the duration of operation was 123.8 ±45.4 minutes in Group-1 and 122.1 ±42.2 minutes in Group-2, and there was no statistically significant difference between the groups (p=0.968). Nephroscopy time was 62.5 ±35.4 minutes and 55.6 ±32.9 minutes in Group-1 and Group-2, respectively. Fluoroscopy time was longer in Group-1 when compared to Group-2 (86.7 ±68.7 seconds vs. 70.9 ±39.5 seconds). Although nephroscopy and fluoroscopy time was longer in Group-1, the difference was not statistically significant (p=0.149 and p=0.342, respectively) (Table 2).

**Table 2:** Comparison of operative data between the study groups *Mean ±standard deviation

Variables	Group-1 (<65 years)	Group-2 (≥65 years)	P-value
Number of patients	139	43	
Duration of operation (minutes)*	123.8 ±45.4	122.1 ±42.2	0.968
Duration of nephroscopy (minutes)*	62.5 ±35.4	55.6 ±32.9	0.149
Duration of fluoroscopy (seconds)*	86.7 ±68.7	70.9 ±39.5	0.342
Access localization			0.479
Lower calyx	84 (60.4%)	23 (53.5%)	
Middle calyx	55 (39.6%)	20 (46.5%)	

When we evaluated the postoperative results, we observed that the amount of hemoglobin drop (the difference between preoperative and first-day postoperative values) was higher in Group-1, which was statistically significant (p=0.001). However, the need for blood transfusion was found to be higher in Group-2 than in Group-1 (18.6% vs. 7.2%, respectively). One patient required blood transfusion in the <65 years group intraoperatively. The length of hospital stay and duration of nephrostomy tube were similar between the two groups (p=0.855 and p=0.352, respectively). The stone-free rate was calculated as 54.7% in Group-1 and 67.4% in Group-2 (p=0.139). Regarding the total complication rates, 34.5% of patients in Group-1 and 46.5% in Group-2 were observed to develop complications (p=0.206). Postoperative complications and related results are shown in detail in Table 3.

**Table 3:** Comparison of complications and postoperative outcomes between the study groups *Mean ±standard deviation ESWL: extracorporeal shock wave lithotripsy; URS: ureterorenoscopy; RIRS: retrograde intrarenal surgery; PCNL: percutaneous nephrolithotomy

Variables	Group-1 (<65 years)	Group-2 (≥65 years)	P-value
Number of patients	139	43	
Overall complication	48 (34.5%)	20 (46.5%)	0.206
Clavien-Dindo classification			
Grade 1/2	31 (22.3%)	18 (41.9%)	0.012
Grade 3/4	17 (12.2%)	2 (4.7%)	0.155
Hemoglobin drop (gr/dl)*	1.9 ±1.3	1.3 ±1.2	0.001
Creatinine change (mg/dl)*	0.2 ±0.2	0.2 ±0.3	0.332
Duration of nephrostomy (days)*	2.3 ±1.0	2.4 ±1.0	0.352
Duration of hospitalization (days)*	4.4 ±2.9	4.1 ±2.1	0.855
Success rate	76 (54.7%)	29 (67.4%)	0.139
Auxiliary procedures			0.222
ESWL	11 (7.9%)	4 (9.3%)	
URS	16 (11.5%)	0	
RIRS	4 (2.9%)	2 (4.7%)	
PCNL	4 (2.9%)	1 (2.3%)	

Examination of the subgroups of complications according to the Clavien scoring system revealed that the rate of minor complications was 22.3% in Group-1 and 41.9% in Group-2 (p=0.012). The major complication rates were determined as 4.7% and 12.2% in Group-1 and Group-2, respectively (p=0.155). In addition, the rate of Clavien grade-2 complications was significantly higher in the elderly group (p=0.019). Ten patients in Group-1 and eight patients in Group-2 required blood transfusion (Clavien class 2), and 15 patients in Group-1 and seven patients in Group-2 experienced fever in the postoperative period (Clavien class 2). Only one patient in Group-1 underwent angioembolization due to the bleeding (Clavien class 3B). Two patients in Group-1 and one in Group-2 required JJ stent insertion under anesthesia due to prolonged urine extravasation (Clavien class 3B) (Table 4).

**Table 4:** Classification of complications according to the Clavien scoring system

Grade	Complication	Group-1 (<65 years; n=139)	Group-2 (≥65 years; n=43)	P-value
0	Total	91 (65.5%)	23 (53.5%)	0.156
1	Postoperative pain that regresses with opioid therapy	2	1	0.482
	Bleeding that does not require a blood transfusion	1	-	
	Postoperative fever that does not require antibiotic change (>38 °C)	3	2	
	Total	6 (4.3%)	3 (6.9%)	
2	Bleeding requiring blood transfusion	10	8	0.019
	Postoperative fever requiring antibiotic change (>38 °C)	15	7	
	Total	25 (17.9%)	15 (34.9%)	
3A	Hydrothorax requiring tube thoracostomy under local anesthesia	2	-	0.106
	Nephrostomy under local anesthesia due to urinoma	1	1	
	Double-J stent insertion under local anesthesia due to urinary leakage from the tract	11	-	
	Total	14 (10.0%)	1 (2.3%)	
3B	Bleeding controlled by angioembolization	1	-	0.948
	Double-J stent placement under general anesthesia due to urinary leakage from the tract	2	1	
	Total	3 (2.1%)	1 (2.3%)	

## Discussion

The increase in the aging population has resulted in more renal stones being detected in geriatric patients. It is important to determine the most appropriate approach when managing renal stones in geriatric patients because of age-related cardiovascular and pulmonary system deterioration and the presence of multiple comorbidities. In elderly patients with multiple comorbidities, observation may be an option for asymptomatic small stones. However, in elderly patients, stone growth is observed to occur over a shorter time, and this causes urinary tract infection, obstruction, and pain that requires analgesics [10], adversely affecting their kidney functions. Although PCNL is accepted as an effective and safe method in large kidney stones, it can result in major complications. A review by Skolarikos et al. stated that the rate of major complications after PCNL was as follows: septicemia (0.9-4.7%), bleeding requiring an intervention (0.6-1.4%), pleural injury (2.3-3.1%), and colonic injury (0.2-0.8%) [11]. Changes in the cardiorespiratory reserve of elderly patients make them less tolerant to bleeding or septic complications [12]. Therefore, a detailed evaluation and a careful approach are required in the management of renal stones in elderly patients.

Despite advances in instrumentation and technology, staghorn stones are still difficult to manage. In a study retrospectively reviewing 42 PCNL procedures performed on 33 patients aged 65 years and older compared with younger patients (47% of the stones were staghorn), 82% (27/33) of the patients were determined to achieve stone-free status or have fragments <5 mm at three months after surgery. In this study, PCNL was shown to be a safe and effective treatment for elderly patients, even in the presence of renal stones; however, a higher rate of transfusion was required in this group [13]. Sahin et al. reported the PCNL results of 27 patients aged over 60 years and compared them to 178 PCNL procedures performed in 166 younger patients during the same timeframe [14]. Only 25% of the patients had staghorn renal stones, and the success rate was 89% and 92% for elderly and younger patients, respectively. In contrast to previous studies reporting higher stone-free rates (78-93%) after PCNL in staghorn renal stones [5,15], the success rates obtained from the current study including only complete staghorn stones were found to be 67.4% and 54.7%, for the elderly and younger groups, respectively. This lower rate of success can be explained by technical limitations, such as the exclusion of partial staghorn stones, use of only one access point for each patient, lithotripsy being performed only with a pneumatic lithotripter, and not using a flexible nephroscope. Similar to our study, Kuzgunbay et al., who performed 47 PCNL procedures in 45 patients aged 65 years with complete staghorn stones and compared their data to 37 younger patients, found the success rate after the first procedure to be 53% in the elderly group and 37.8% in the control group [16].

In our study, in which only complete staghorn stones were included, the stone sizes were similar between elderly and younger patients, which shows the comparability of the two groups in terms of stone burden. Furthermore, the length of hospital stay, operation and fluoroscopy durations, and success rate were found to be similar in the elderly and younger groups. Therefore, we consider that advanced age does not have a negative effect on intraoperative parameters and postoperative outcomes in complex stones. However, in our study, while the decrease in hemoglobin was significantly higher in younger people (1.9 ±1.3 g/dl vs. 1.3 ±1.2 g/dl, p=0.001), the rate of transfusion requirement was moderately higher in the elderly (7.2% vs. 18.6%). This suggests that the rate of transfusion was higher in the elderly relative to the decrease in hemoglobin. Stoller et al. found higher blood transfusion rates after PCNL in elderly patients with complex renal stones [13]. Sahin et al. reported the transfusion rates after PCNL of 21% in elderly patients and 18% in younger patients [14]. In another study, the transfusion rate after PCNL in staghorn renal stones was detected to be 10.6% in the elderly and 13.5% in the younger group, while the hemoglobin change was 1.46 ±1.29 g/dl and 1.70 ±1.33 g/dl, respectively [16].

PCNL is recognized as an effective and safe treatment modality for large kidney stones. Although the efficacy of the procedure has been proven, complication rates of up to 83% have been reported in the literature, including bleeding requiring transfusion (7%), organ damage (0.4%), and infectious events (up to 33%) [17,18]. Elderly patients tend to have more comorbidities, making them more vulnerable to fatal bleeding and septic complications [19]. In a study by Okeke et al., the overall complication rate after PCNL was significantly higher in elderly patients [20]. However, Karami et al. reported that age alone was not a predictive factor for high complication rates [21]. In another study, no major complications were observed after PCNL in staghorn renal stones in the elderly and younger populations, and their minor complication rates were also similar [16]. In contrast, in our study, there was a higher rate of minor complications, such as bleeding requiring transfusion, in eight and postoperative fever requiring an antibiotic change in seven of the 43 patients in the elderly group, while sepsis, pneumothorax, and bowel injury were not observed in either group. This suggests that elderly patients are less tolerant of bleeding and less resistant to infectious events because they are more prone to having comorbidities. To our knowledge, this is the first study to separately evaluate post-PCNL complications in elderly patients with complete staghorn renal stones according to the Clavien-Dindo classification.

There are some limitations to our study. Firstly, it had a retrospective design and a limited number of patients. Secondly, there was no long-term comparison of surgical complications. Hence, further prospective studies are needed with a larger series of geriatric patients with staghorn stones, focusing specifically on their medical complications.

## Conclusions

This study showed that PCNL could be effectively and safely applied in the elderly population for the treatment of complete staghorn renal stones. However, since minor complications after PCNL are seen at a higher rate in this patient group, it is necessary to be more cautious in terms of possible complication development.

## References

[1] Woitok BK, Ravioli S, Funk GC, Lindner G (2021). Characteristics of very elderly patients in the emergency department - a retrospective analysis. Am J Emerg Med.

[2] Ganpule AP, Desai M (2008). Management of the staghorn calculus: multiple-tract versus single-tract percutaneous nephrolithotomy. Curr Opin Urol.

[3] Torricelli FC, Monga M (2020). Staghorn renal stones: what the urologist needs to know. Int Braz J Urol.

[4] Wollin DA, Preminger GM (2018). Percutaneous nephrolithotomy: complications and how to deal with them. Urolithiasis.

[5] Desai M, Jain P, Ganpule A, Sabnis R, Patel S, Shrivastav P (2009). Developments in technique and technology: the effect on the results of percutaneous nephrolithotomy for staghorn calculi. BJU Int.

[6] Desai M, De Lisa A, Turna B (2011). The clinical research office of the endourological society percutaneous nephrolithotomy global study: staghorn versus nonstaghorn stones. J Endourol.

[7] Al-Kohlany KM, Shokeir AA, Mosbah A (2005). Treatment of complete staghorn stones: a prospective randomized comparison of open surgery versus percutaneous nephrolithotomy. J Urol.

[8] Dindo D, Demartines N, Clavien PA (2004). Classification of surgical complications: a new proposal with evaluation in a cohort of 6336 patients and results of a survey. Ann Surg.

[9] Tiselius HG, Andersson A (2003). Stone burden in an average Swedish population of stone formers requiring active stone removal: how can the stone size be estimated in the clinical routine?. Eur Urol.

[10] Burgher A, Beman M, Holtzman JL, Monga M (2004). Progression of nephrolithiasis: long-term outcomes with observation of asymptomatic calculi. J Endourol.

[11] Skolarikos A, de la Rosette J (2008). Prevention and treatment of complications following percutaneous nephrolithotomy. Curr Opin Urol.

[12] Tonner PH, Kampen J, Scholz J (2003). Pathophysiological changes in the elderly. Best Pract Res Clin Anaesthesiol.

[13] Stoller ML, Bolton D, St Lezin M, Lawrence M (1994). Percutaneous nephrolithotomy in the elderly. Urology.

[14] Sahin A, Atsü N, Erdem E, Oner S, Bilen C, Bakkaloğlu M, Kendi S (2001). Percutaneous nephrolithotomy in patients aged 60 years or older. J Endourol.

[15] Soucy F, Ko R, Duvdevani M, Nott L, Denstedt JD, Razvi H (2009). Percutaneous nephrolithotomy for staghorn calculi: a single center's experience over 15 years. J Endourol.

[16] Kuzgunbay B, Turunc T, Yaycioglu O (2011). Percutaneous nephrolithotomy for staghorn kidney stones in elderly patients. Int Urol Nephrol.

[17] Michel MS, Trojan L, Rassweiler JJ (2007). Complications in percutaneous nephrolithotomy. Eur Urol.

[18] Cracco CM, Scoffone CM (2011). ECIRS (endoscopic combined intrarenal surgery) in the Galdakao-modified supine Valdivia position: a new life for percutaneous surgery?. World J Urol.

[19] Akman T, Binbay M, Ugurlu M (2012). Outcomes of retrograde intrarenal surgery compared with percutaneous nephrolithotomy in elderly patients with moderate-size kidney stones: a matched-pair analysis. J Endourol.

[20] Okeke Z, Smith AD, Labate G (2012). Prospective comparison of outcomes of percutaneous nephrolithotomy in elderly patients versus younger patients. J Endourol.

[21] Karami H, Mazloomfard MM, Golshan A, Rahjoo T, Javanmard B (2010). Does age affect outcomes of percutaneous nephrolithotomy?. Urol J.

